# Multicenter comparison of analytical interferences of 25-OH vitamin D immunoassay and mass spectrometry methods by endogenous interferents and cross-reactivity with 3-epi-25-OH-vitamin D_3_

**DOI:** 10.1016/j.plabm.2023.e00347

**Published:** 2023-12-12

**Authors:** Joon Hee Lee, Jong Do Seo, Kyunghoon Lee, Eun Youn Roh, Yeo-Min Yun, Yong-Wha Lee, Sung-Eun Cho, Junghan Song

**Affiliations:** a Department of Laboratory Medicine, Seoul National University College of Medicine, Seoul, South Korea; b Department of Laboratory Medicine, Seoul National University Bundang Hospital, Seongnam, South Korea; c Department of Laboratory Medicine, Konkuk University School of Medicine, Seoul, South Korea; d Department of Laboratory Medicine, Seoul Metropolitan Government-Seoul National University Boramae Medical Center, Seoul, South Korea; e Department of Laboratory Medicine & Genetics, Soonchunhyang University Bucheon Hospital, Bucheon, South Korea; f Department of Endocrine Substance Analysis Center (ESAC), Green Cross Laboratories (GC Labs), Yongin, South Korea

**Keywords:** Vitamin D, Analytical interference, Immunoassay, Mass spectrometry, 3-epi-25-OH-Vitamin D_3_

## Abstract

**Background:**

Vitamin D (vit-D) deficiency is highly prevalent in the Korean population, highlighting the need for accurate measurements. In this study, the interferences by endogenous and cross-reactive substances were compared between routine vit-D immunoassays and mass spectrometry (MS) methods.

**Methods:**

Two MS methods and 4 immunoassays from different manufacturers (Abbott, Beckman Coulter, Roche, Siemens) were compared. Residual samples that were icteric, lipemic, hemolyzed, high in rheumatoid factor, from myeloma patients, or patients undergoing hemodialysis were collected. Also, 4 levels of National Institute of Standards and Technology (NIST) Standard Reference Material 972a, and 12 samples serially spiked with 3-epi-25-OH-D_3_ were prepared.

**Results:**

Significant interferences were observed in hemolytic (Roche), icteric (Beckman and Siemens) and lipemic samples (all 4 immunoassays). Level 4 NIST material and 3-epi-25-OH-D_3_-spiked samples induced significant cross-reactivity, yielding higher total vit-D measurements in non-epimer-separating MS methods, and both the Beckman and Roche immunoassays.

**Conclusion:**

Most observed interferences were consistent with manufacturers’ claims, but overall improvement of immunoassay bias limits is required. Awareness of potential interference is important to increase the accuracy of vit-D measurements. Moreover, care is due when interpreting vit-D results of newborns, infants and less commonly, pregnant women, who are known to have physiologically high levels of the highly cross-reactive 3-epi-25-OH-D_3_.

## Highlights

• Endogenous and cross-reactive substances can cause erroneous vitamin D results.
• Generally, overall improvement of immunoassay bias limits is required.
• Hemolytic, icteric, or lipemic samples caused large errors in immunoassays.
• 3-epi-25-OH-D_3_-separating mass spectrometry methods should be encouraged.
• Epimer interference must be considered in vitamin D results of pediatric samples.

## Abbreviations

Vit-D: Vitamin D
MS: mass spectrometry
NIST: National Institute of Standards and Technology
25-OH-D: 25-OH-vitamin D
LC-MS/MS: liquid chromatography-tandem mass spectrometry
RMP: reference measurement procedure
DEQAS: Vitamin D External Quality Assessment Scheme
NIH: National Institutes of Health
VitDQAP: Vitamin D Metabolites Quality Assurance Program
CDC: Centers for Disease Control and Prevention
VDSCP: Vitamin D Standardization Certification Program
VDSP: Vitamin D Standardization Program
SRM: Standard Reference Material
LDT: laboratory-developed test

## 1 Introduction

Vitamin D is a cholesterol-derived prohormone obtained from either ultraviolet-B exposure and dietary sources [1]. The importance of vitamin D has taken the spotlight for several years, with it known to play an important role in calcium homeostasis and bone metabolism [2]. Vitamin D deficiency is associated with a wide range of diseases including rickets, arthritis, preeclampsia, various cardiovascular, renal, autoimmune diseases, and cancer [3,4]. Depending on the intake source, vitamin D exists as either vitamin D_3_ (cholecalciferol) or vitamin D_2_ (ergocalciferol) in the human body. Upon metabolism, most vitamin D in the prohormone state is converted to 25-OH vitamin D (25-OH-D), the main form in systemic circulation with a long half-life. Further hydroxylation will convert 25-OH-D to either the biologically active 1,25-(OH)_2_-D, or the inactive 24,25-(OH)_2_-D. Due to the low concentration and short half-life of 1,25-(OH)_2_-D, total 25-OH-D is the analyte-of-choice commonly measured by clinical vitamin D assays [5].

In clinical laboratories, immunoassays are the most common method for analyzing 25-OH-D, followed by liquid chromatography-tandem mass spectrometry (LC-MS/MS). The high specificity attainable with LC-MS/MS often makes it the technique-of-choice for reference measurement procedures (RMP), and the current candidate RMPs for 25-OH-D utilize isotope-dilution liquid chromatography-tandem mass spectrometry [[6], [7], [8]]. Despite the analytical advantages of LC-MS/MS, most laboratories in Korea use 25-OH-D immunoassays due to various practical issues. Compared to MS-based assays, 25-OH-D immunoassays are more vulnerable to interference from endogenous interferents [9,10] and show cross-reactivity with various substances such as 1,25-(OH)_2_-D_3_, 24,25-(OH)_2_-D_3_, and 3-epi-25-OH-Vitamin D_3_ (3-epi-25-OH-D_3_) [1,11].

To improve the accuracy of 25-OH-D assays, international quality assessment programs such as the Accuracy-based 25-OH-D CAP survey, Vitamin D External Quality Assessment Scheme (DEQAS), and the National Institute of Standards and Technology (NIST)/National Institutes of Health (NIH) Vitamin D Metabolites Quality Assurance Program (VitDQAP) have been implemented worldwide. Also, the Centers for Disease Control and Prevention (CDC) regularly publishes a certified list under the Vitamin D Standardization Certification Program (VDSCP), consisting of 25-OH-D assays which meet analytical performance criteria of bias ≤5% and imprecision ≤10% using commutable standard materials [12]. Despite these efforts, study results published by DEQAS [11] and Vitamin D Standardization Program (VDSP) [13] have identified several limitations with common 25-OH-D assays, especially with regards to 3-epi-25-OH-D_3_ cross-reactivity.

Although previous studies have evaluated the effects of endogenous interferents or cross-reactivity, they are limited to individual assays. In this multicenter study, the analytical interference of four routinely used commercial immunoassays and two MS assays, by endogenous interferents and cross-reactivity with 3-epi-25-OH-D_3_ was simultaneously compared.

## 2 Methods

### 2.1 Samples

This study acquired the approval of the institutional review board(s) (IRB No. SNUBH X-2302-813-901**,** KUMC 2021-10-032). Residual samples with volumes ≥1.6 mL were collected from participating institutes in VACUETTE® serum separating tubes (Greiner AG, Kremsmünster, Austria). For inter-method comparison between reference MS methods, 150 samples were collected in accordance with Clinical & Laboratory Standards Institute (CLSI) guideline EP09-A3 [14], consisting of 3 groups of 50 samples each with 25-OH-D concentrations of 5–15, 15–25, and >25 ng/mL respectively. For evaluation of interference via endogenous substances, 45 samples were collected, consisting of 5 hemolyzed samples, 5 icteric samples (total bilirubin >20 mg/dL), 5 lipemic samples (triglyceride >500 mg/dL), 10 myeloma patient samples (paraprotein >1.1 g/dL), 10 samples with rheumatoid factor >200 IU/mL, and 10 samples from hemodialysis patients. All samples were aliquoted and stored at −80 °C until measurement.

### 2.2 Standard materials

Four levels of NIST Standard Reference Material (SRM) 972a (National Institute of Standards & Technology, Gaithersburg, MD, USA) were prepared, with each level having different compositions and concentrations of vitamin D (Supplementary Table S1). Each SRM 972a level consisted of frozen human serum with different assigned 25-OH-D values, with levels 1–3 consisting of normal human pooled serum, and level 4 consisting of spiked human serum to enrich 3-epi-25-OH-D_3_ (level 4) [15].

### 2.3 Serially spiked residual samples for 3-epi-25-OH-D_3_

Three residual samples of 25-OH-D concentrations near 20, 30, and 50 ng/mL were prepared. After aliquoting, each sample was spiked with 3-epi-25-OH-D_3_ (Sigma-Aldrich, Burlington, MO, USA) dissolved in absolute ethanol, to prepare 4 levels of each original sample with 0, 10, 25, and 50% spiking concentrations. For example, a 4-level spiking set of a 20 ng/mL sample would be prepared by spiking with 0, 2, 5 and 10 ng/mL 3-epi-25-OH-D_3_ to make 0, 10, 25 and 50% spiking concentrations respectively. Total 25-OH-D results measured by the MS2E-method (see ’25-OH-D assays’ below for details) were used as reference values during evaluation of 3-epi-25-OH-D_3_ interference.

### 2.4 25-OH-D assays

Two MS based methods and 4 commercial immunoassays, including Abbott Alinity 25-OH Vitamin D with Alinity i system (Abbott; Abbott Laboratories, Abbott Park, IL, USA), Beckman Coulter Access 25-OH Vitamin D Total with Beckman coulter Access immunoassay analyzer (Beckman; Beckman Coulter, Brea, CA, USA), Roche Elecsys Vitamin D Total II with Cobas 800 e801 analyzer (Roche; Roche Diagnostics GmbH, Mannheim, Germany), and Siemens Atellica IM Vitamin D Total assay with Atellica IM analyzer (Siemens; Siemens Healthineers, Forchheim, Germany), were evaluated. With the exception of the Beckman immunoassay, all other 3 immunoassays are certified assays approved via the CDC VDSCP [12]. The analytical measurement ranges and imprecision data provided for the 4 immunoassays are shown in Table 1. The provided imprecision data was allegedly obtained according to protocols from CLSI guidelines EP05-A3 [16] or A2 [17]. Both MS based methods are laboratory-developed tests (LDT), with the protocol for one method (MS1) utilizing Diels-Alder derivatization previously published [18], with some changes in instrument to a Nexera X2 Series HPLC/UHPLC (Shimadzu, Kyoto, Japan) and SCIEX Triple Quad 6500+ LC-MS/MS System (Sciex.

**Table 1:** Analytical measurement range and imprecision of 4 commercial immunoassays.

Assay	AMR^a^ (ng/mL)	Concentration of evaluated sample (ng/mL)	Repeatability		Within-laboratory	
			SD (ng/mL)	CV %)	SD (ng/mL)	CV (%)
Abbott	3.5–154.2	20.6	0.57	2.8	0.67	3.3
Beckman	7.0–120.0	13.3	0.50	3.8	1.00	7.7
Roche	3.0–100.0	9.83	0.88	8.9	1.06	10.8
Siemens	4.2–150.0	10.75	0.84	N/A^b^	1.42	N/A^b^

a Abbreviations: AMR, analytical measurement range; SD, standard deviation; CV, coefficient of variation.

b Not applicable.

Framingham, MA, USA) conducted at Seoul National University Bundang Hospital. The second method (MS2), conducted at Green Cross Laboratories, is also a LDT but follows the protein precipitation, solvent extraction and derivatization steps using reagents from a PerkinElmer MSMS Vitamin D kit (PerkinElmer, Turku, Finland). MS2 utilizes an ACQUITY UPLC I-Class System (Waters, Milford, MA, USA) equipped with a Waters ACQUITY UPLC BEH C18 column (2.1 × 50 mm, 1.7 μm; Waters, Milford, MA, USA) on a Xevo TQD (Waters, Milford, MA, USA) MS/MS system.

The routine MS methods (MS1 and MS2) cannot separate 3-epi-25-OH-D_3,_ thus including 3-epi-25-OH-D_3_ in the total 25-OH-D results. For evaluation of 3-epi-25-OH-D_3_ cross-reactivity, another method (MS2E-), also conducted at Green Cross Laboratories, was utilized. MS2E-follows the same preparation protocol as MS2, but instead uses a Nexera-X2-LC-30AD UPLC (Shimadzu, Tokyo, Japan) system equipped with a Kinetex XB C18 column (2.1 × 150 mm, 2.6 μm; Phenomenex, Torrance, CA, USA) on a Triple Quad 4500MD (Sciex, Framingham, MA, USA) MS/MS system with different analysis conditions to MS2, and has a longer runtime of 16 min (compared to 4.5 min for MS2).

Also, 24,25-(OH)_2_-D_3_ was measured via LC-MS/MS at Seoul National University Bundang Hospital. The immunoassays were conducted at 3 different institutes, with the Beckman test conducted at Soonchunhyang University Bucheon Hospital, and the remaining 3 conducted at Konkuk University Medical Center, except for epimer spiked samples using the Abbott test, which was conducted at Boramae Medical Center. All 25-OH-D measurements are reported in conventional units (ng/mL), with 1 ng/ml corresponding to a SI unit of 2.5 nmol/L [19].

### 2.5 Evaluation of interference due to endogenous interferents

Measurements of 25-OH-D (25-OH-D2 + 25-OH-D3 + any endogenous 3-epi-25-OH-D3) were conducted via MS1, MS2, and the 4 commercial immunoassays from the collected samples with (a) hemolysis, (b) bilirubin, (c) lipids, (d) myeloma (paraprotein), (e) rheumatoid factor, and (f) hemodialysis. The mean values of MS1 and MS2 are used as reference values to compare with the immunoassays, and absolute and percentage differences were calculated.

### 2.6 Evaluation of interference due to 3-epi-25-OH-D_3_ – level 4 NIST SRM 972a material

Measurements of 25-OH-D (25-OH-D2 + 25-OH-D3 + any existing 3-epi-25-OH-D3) were conducted via MS1, MS2, and the 4 commercial immunoassays. The mean values of MS1 and MS2 are used as reference values to compare with the immunoassays.

### 2.7 Evaluation of interference due to 3-epi-25-OH-D_3_ – serially spiked samples

Measurements of 25-OH-D (25-OH-D2 + 25-OH-D3 + any existing 3-epi-25-OH-D3) were conducted via MS1, MS2, MS2E-, and the 4 commercial immunoassays. The MS2E-values are used as reference values to compare with MS1, MS2, and the immunoassays, and absolute and percentage differences were calculated.

### 2.8 Interference limits supplied by immunoassay manufacturers

The 10% bias limits inducible by endogenous interferents are shown in Supplementary Table S2. The cross-reactivity (%) of various 25-OH-D subtypes are shown in Supplementary Table S3.

### 2.9 Statistical analysis

Passing-Bablok regression was used to compare MS methods and prove analytical equivalence. Mean values of the MS methods were used as reference values to compare results with other immunoassays. Multiple regression was used to evaluate effects of changes in endogenous interferent concentrations/grades. For interference evaluation of 3-epi-25-OH-D_3_ spiked samples, the mean biases (%) compared to reference results measured by the epimer-separating MS2E-method were compared between methods. All statistical analyses were performed using MedCalc version 20.210 (MedCalc Software, Mariakerke, Belgium).

## 3 Results

### 3.1 Comparison between MS methods

**Fig. 1:**
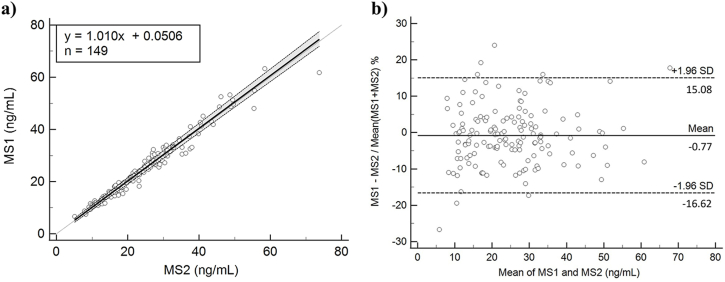
Passing-Bablok regression (a) and Bland-Altman plot (b) showing comparison between MS methods.

From 149 measurements (1 sample excluded from analysis due to insufficient volume), Passing-Bablok regression (Fig. 1) between MS methods (MS1 vs MS2) yielded a slope (95% confidence interval; CI) of 1.010 (0.983–1.042), intercept of 0.051 (from −0.587 to 0.650), and Spearman rank correlation coefficient of 0.983 (*P* < 0.01). Bland-Altman plot of the two MS methods (Fig. 1) showed a mean inter-method bias of −0.77% with ±1.96 SD lower/upper limits of −16.62/15.08%.

### 3.2 Recovery study

**Fig. 2:**
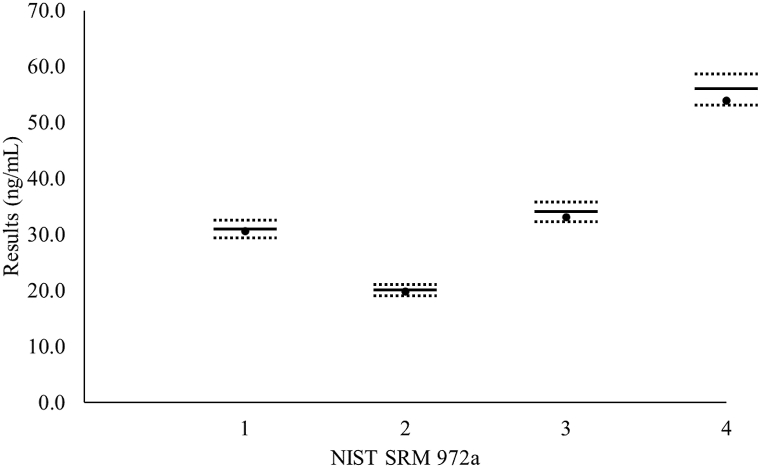
Four levels of NIST SRM 972a mean values measured by both MS methods (black dots). The solid and dashed lines for each level represent the assigned value and allowable limits (±5%) assigned according to VDSCP criteria, respectively. Assigned values are the sum of 25-OH-D_3_, 25-OH-D_2_ in levels 1–3, and obtained values are the sum of 25-OH-D_3_, 25-OH-D_2_, and 3-epi-25-OH-D_3_.

Four levels of NIST SRM 972a standard material were measured by both MS methods, and mean values are depicted in Fig. 2. Measured MS mean total 25-OH-D values were within the VDSCP 5% bias limits from the assigned values (sum of 25-OH-D_3_, 25-OH-D_2_) for levels 1–3 NIST SRM 972a material, and the obtained value (sum of 25-OH-D_3_, 25-OH-D_2_, and 3-epi-25-OH-D_3_) of level 4 NIST SRM 972a material, assuming that 3-epi-25-OH-D_3_ is included in the total 25-OH-D measurements.

### 3.3 Interference due to endogenous interferents

**Fig. 3:**
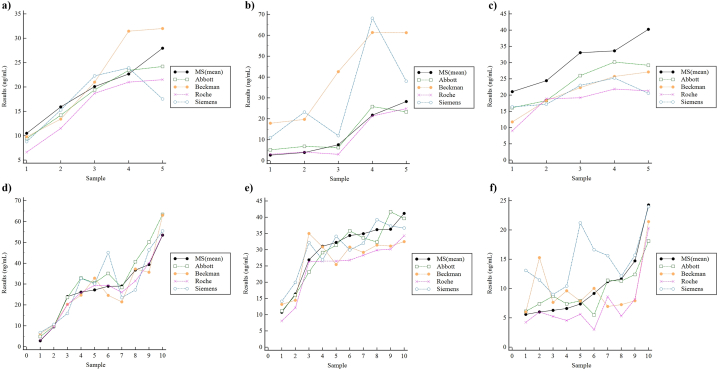
**(Color).** Evaluation of interference due to endogenous interferents: (a) hemolysis, (b) bilirubin, (c) lipids, (d) myeloma (paraprotein), (e) rheumatoid factor, and (f) hemodialysis.

25-OH-D measurements in hemolyzed, icteric, lipemic samples, or from myeloma patients, rheumatoid factor (RF) > 200 IU/mL, and samples from patients undergoing hemodialysis are depicted in Fig. 3 and Table 2. In hemolytic samples, immunoassay measurements of some individual samples showed biases >30% compared to MS values, but no significant interference pattern was observed, except for the Roche assay, which showed a consistent negative bias (from 6.7 to 36.8%) in all samples. In icteric samples, positive biases compared to MS values were observed in all sample measurements with the Beckman and Siemens assays, with % bias ranges of 116.2–591.5% and 34.4–497.4% respectively. In lipemic samples, a general negative bias (approximately 20–30%) compared to MS values was observed in all 4 immunoassays. In myeloma patient samples, the Abbott assay showed the most positive bias (8/10 samples), with 7 of these samples giving biases >10%. In samples with high RF, no significant interference pattern was observed, except for the Roche assay, which showed a consistent negative bias (from 2.2 to 26.2%) in all samples. In hemodialysis samples, negative biases (from 16.7 to 67.2%) were again observed in all but one of the Roche assay results, positive biases were observed in all but one of the Siemens assay results, and intermittent biases exceeding >100% were observed in the Beckman and Siemens assay results. In some samples, the measured MS values were below the analytical measurement range of the commercial immunoassays. Multiple regression showed no significant effect due to changes in endogenous interferent concentrations or grades (data not shown).

**Table 2:** 25-OH-D measurements of MS and immunoassays in presence of endogenous interferents (absolute and percentage differences of immunoassays compared to MS(mean) results).

(a) Hemolysis															
Sample	Sex	Age	H index	24,25-(OH)_2_-D_3_ (ng/mL)	MS1 (ng/mL)	MS2 (ng/mL)	MS (mean) (ng/mL)	Abbott (ng/mL)	(% diff)	Beckman (ng/mL)	(% diff)	Roche (ng/mL)	(% diff)	Siemens (ng/mL)	(% diff)
1	F	83	2	0.19	10.3	10.7	10.5	9.4	(-10.6)	9.8	(-6.9)	6.6	(-36.8)	8.8	(-16.2)
2	F	72	1	0.88	15.3	16.6	15.9	14.2	(-10.9)	13.4	(-15.8)	11.5	(-27.8)	15.3	(-3.9)
3	F	74	2	1.40	19.7	20.4	20.1	19.4	(-3.4)	21.0	(4.7)	18.7	(-6.8)	22.3	(11.1)
4	F	66	3	2.26	22.1	23.3	22.7	23.4	(3.2)	31.4	(38.7)	21.0	(-7.4)	23.9	(5.5)
5	F	66	4	2.00	28.5	27.4	28.0	24.2	(-13.5)	32.0	(14.5)	21.5	(-23.1)	17.5	(-37.3)

(b) Bilirubin															
Sample	Sex	Age	T. Bil (mg/dL)	24,25-(OH)_2_-D_3_ (ng/mL)	MS1 (ng/mL)	MS2 (ng/mL)	MS (mean) (ng/mL)	Abbott (ng/mL)	(% diff)	Beckman (ng/mL)	(% diff)	Roche (ng/mL)	(% diff)	Siemens (ng/mL)	(% diff)
1	M	84	29.9	0.05	1.9	3.2	2.6	5.2	(100.8)	17.9	(591.5)	3.0	(15.8)	11.0	(323.6)
2	M	67	22.9	0.36	3.9	3.9	3.9	6.8	(74.6)	19.7	(406.0)	4.0	(3.7)	23.3	(497.4)
3	F	66	22.5	0.64	7.2	7.9	7.6	6.2	(-18.0)	42.6	(462.5)	3.0	(-60.3)	12.0	(58.6)
4	F	71	25.2	1.85	20.9	22.8	21.8	25.9	(18.7)	61.3	(180.9)	21.4	(-1.9)	68.1	(212.1)
5	M	65	33.4	2.51	27.4	29.2	28.3	23.3	(-17.7)	61.2	(116.2)	24.9	(-12.0)	38.0	(34.4)

(c) Lipids															
Sample	Sex	Age	TG (mg/dL)	24,25-(OH)_2_-D_3_ (ng/mL)	MS1 (ng/mL)	MS2 (ng/mL)	MS (mean) (ng/mL)	Abbott (ng/mL)	(% diff)	Beckman (ng/mL)	(% diff)	Roche (ng/mL)	(% diff)	Siemens (ng/mL)	(% diff)
1	M	29	612	1.34	20.8	21.4	21.1	16.1	(-23.6)	11.8	(-44.2)	9.0	(-57.4)	16.4	(-22.2)
2	M	47	575	1.48	24.6	24.3	24.4	18.3	(-25.0)	18.3	(-25.0)	18.9	(-22.6)	17.2	(-29.7)
3	M	ND	1510	0.12	31.2	35.0	33.1	26.0	(-21.4)	22.3	(-32.6)	19.2	(-41.9)	23.0	(-30.5)
4	M	42	794	2.92	34.8	32.6	33.7	30.2	(-10.3)	25.7	(-23.5)	21.9	(-34.9)	25.3	(-24.9)
5	F	50	550	2.04	45.6	35.0	40.3	29.2	(-27.5)	27.1	(-32.6)	21.3	(-47.1)	20.6	(-49.0)

(d) Myeloma (paraprotein)																
Sample	Sex	Age	M-protein (g/dL)	Total protein (g/dL)	24,25-(OH)_2_-D_3_ (ng/mL)	MS1 (ng/mL)	MS2 (ng/mL)	MS (mean) (ng/mL)	Abbott (ng/mL)	(% diff)	Beckman (ng/mL)	(% diff)	Roche (ng/mL)	(% diff)	Siemens (ng/mL)	(% diff)
1	M	77	2.17	8.2	0.09	2.0	3.7	2.8	4.9	(74.1)	6.2	(121.0)	3.1	(10.8)	6.8	(141.6)
2	F	67	2.06	7.3	0.64	9.2	9.8	9.5	9.8	(3.4)	10.4	(10.1)	9.6	(0.7)	10.7	(12.3)
3	F	86	2.52	7.4	1.24	25.8	22.3	24.0	23.6	(-1.8)	20.4	(-15.3)	20.1	(-16.4)	16.0	(-33.6)
4	F	80	1.89	11.4	2.38	26.6	25.6	26.1	32.9	(26.0)	24.7	(-5.6)	26.0	(-0.4)	32.8	(25.5)
5	M	80	5.62	8.4	1.5	26.6	27.8	27.2	30.6	(12.6)	32.9	(20.9)	29.5	(8.5)	30.1	(10.8)
6	M	49	6.64	9.1	4.43	28.9	29.1	29.0	35.1	(21.0)	24.6	(-15.3)	29.4	(1.4)	45.1	(55.5)
7	M	78	2.43	7.5	1.79	28.4	29.7	29.0	28.4	(-2.2)	21.4	(-26.1)	26.1	(-10.1)	23.5	(-19.1)
8	M	76	3.00	9.6	1.83	36.7	36.9	36.8	40.7	(10.7)	37.2	(1.2)	31.5	(-14.3)	27.2	(-26.0)
9	M	66	2.39	13.6	1.28	43.7	35.0	39.3	50.2	(27.6)	35.7	(-9.3)	40.1	(2.0)	46.4	(17.9)
10	F	86	3.22	8.8	4.34	56.3	50.7	53.5	63.4	(18.5)	63.0	(17.7)	53.7	(0.4)	55.5	(3.7)

(e) Rheumatoid Factor															
Sample	Sex	Age	RF^a^ (IU/mL)	24,25-(OH)_2_-D_3_ (ng/mL)	MS1 (ng/mL)	MS2 (ng/mL)	MS (mean) (ng/mL)	Abbott (ng/mL)	(% diff)	Beckman (ng/mL)	(% diff)	Roche (ng/mL)	(% diff)	Siemens (ng/mL)	(% diff)
1	M	84	546.4	0.36	10.5	11.0	10.7	11.1	(3.3)	13.2	(22.8)	8.1	(-25.0)	14.1	(31.3)
2	M	46	>650.0**	0.38	16.5	16.3	16.4	16.0	(-2.4)	14.4	(-12.1)	12.1	(-26.2)	19.9	(21.6)
3	F	68	842.7	0.85	26.8	27.0	26.9	23.1	(-14.1)	35.0	(30.2)	26.3	(-2.2)	32.2	(19.6)
4	F	55	480.8	3.09	30.1	32.0	31.1	29.1	(-6.3)	30.9	(-0.6)	26.5	(-14.7)	26.9	(-13.4)
5	M	55	488.4	2.37	30.6	34.0	32.3	31.4	(-2.7)	25.5	(-21.1)	26.5	(-17.9)	34.2	(5.8)
6	M	62	586.5	3.11	33.1	35.6	34.4	35.8	(4.2)	30.8	(-10.3)	26.8	(-22.0)	30.0	(-12.8)
7	F	69	220.0	2.92	36.5	33.4	35.0	33.6	(-3.9)	29.2	(-16.4)	28.3	(-19.0)	32.0	(-8.4)
8	F	69	739.5	3.08	35.3	37.2	36.2	32.4	(-10.5)	31.4	(-13.2)	29.9	(-17.4)	39.3	(8.4)
9	F	66	612.8	3.69	35.5	37.2	36.3	41.7	(14.8)	31.2	(-14.3)	30.2	(-16.9)	37.3	(2.6)
10	F	78	772.4	2.63	41.3	41.1	41.2	39.6	(-3.9)	32.5	(-21.0)	34.3	(-16.7)	36.7	(-10.9)

(f) Hemodialysis														
Sample	Sex	Age	24,25-(OH)_2_-D_3_ (ng/mL)	MS1 (ng/mL)	MS2 (ng/mL)	MS (mean) (ng/mL)	Abbott (ng/mL)	(% diff)	Beckman (ng/mL)	(% diff)	Roche (ng/mL)	(% diff)	Siemens (ng/mL)	(% diff)
1	F	85	0.17	4.5	6.7	5.6	6.1	(8.9)	6.0	(7.3)	4.2	(-24.3)	13.1	(133.8)
2	M	64	0.48	4.7	7.3	6.0	7.4	(23.0)	15.3	(154.2)	6.0	(0.1)	11.5	(90.5)
3	M	81	0.18	5.3	7.2	6.3	8.7	(38.5)	7.6	(21.7)	5.2	(-16.7)	9.0	(43.0)
4	M	55	0.14	5.6	7.6	6.6	7.4	(12.0)	9.6	(45.4)	4.6	(-31.0)	10.4	(57.5)
5	M	95	0.37	6.5	8.2	7.4	7.9	(7.3)	7.9	(6.7)	5.6	(-23.6)	21.2	(188.2)
6	M	92	0.23	12.8	5.5	9.2	5.5	(-39.9)	10.0	(9.5)	3.0	(-67.2)	16.6	(81.7)
7	M	48	0.12	11.0	11.4	11.2	11.4	(2.0)	7.0	(-37.7)	8.6	(-23.0)	15.6	(40.0)
8	M	48	0.21	10.8	12.4	11.6	11.3	(-2.8)	7.2	(-37.7)	5.3	(-54.3)	12.1	(4.3)
9	F	44	0.33	14.3	15.1	14.7	12.4	(-15.7)	7.9	(-46.2)	8.2	(-44.3)	15.7	(6.8)
10	F	75	0.42	24.9	23.6	24.3	18.1	(-25.4)	21.4	(-11.7)	20.3	(-16.3)	24.0	(-1.2)

*Abbreviations: TG, triglycerides; ND, no data.**Insufficient sample volume for retesting after dilution.

a Abbreviations: TG, triglycerides; ND, no data.

### 3.4 Interference due to 3-epi-25-OH-D_3_ – level 4 NIST SRM 972a material

**Fig. 4:**
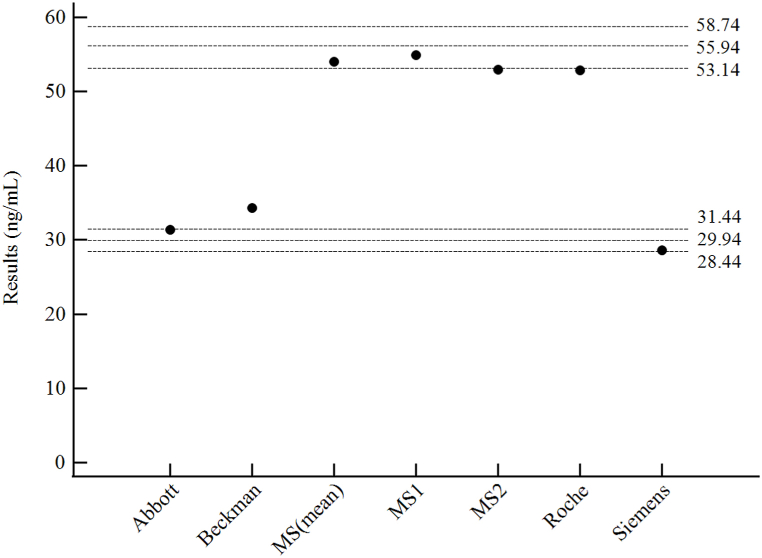
Measurements of the level 4 NIST SRM 972a material with each assay. The top 3 horizontal dashed lines represent the assigned value (2nd line) including 3-epi-25-OH-D_3_ and ±5% bias limits (1st and 3rd lines). The bottom 3 dashed lines represent the assigned value (5th line) excluding 3-epi-25-OH-D_3_, and ±5% bias limits (4th and 6th lines).

Level 4 NIST SRM 972a material measurements are shown in Fig. 4. Both MS methods and the Roche assay showed high-cross reactivity with 3-epi-25-OH-D_3_ in the NIST material, yielding high total 25-OH-D measurements. The Abbott and Siemens assays did not show cross-reactivity with 3-epi-25-OH-D_3_ and gave measurement results within VDSCP-recommended 5% bias limits. The Beckman assay showed minimal cross-reactivity with 3-epi-25-OH-D_3_, but the measurement result exceeded the 5% bias limits.

### 3.5 Interference due to 3-epi-25-OH-D_3_ – serially spiked samples

**Fig. 5:**
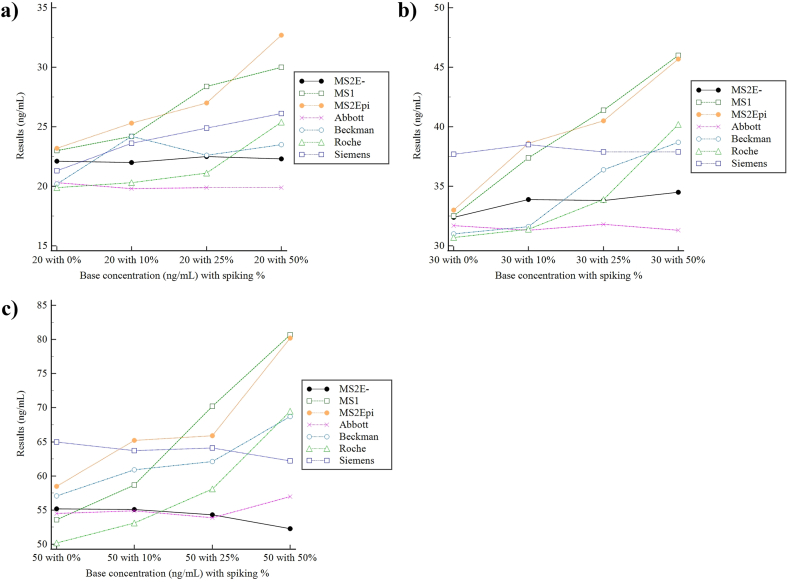
**(Color).** Measurements of the serially spiked samples (0–50%) with each assay near concentrations of (a) 20 ng/mL, (b) 30 ng/mL, and (c) 50 ng/mL. The black line depicts the epimer separating MS method (MS2E-), which is used as the reference method. Base 25-OH-D concentrations and spiking concentrations are depicted on the x-axis as [base concentration/spiking%]. MS2 is depicted as “MS2Epi” in the figure to highlight the inclusion of 3-epi-25-OH-D_3_ in its measurement.

Measurements of the 12 spiked samples are shown in Fig. 5 and Supplementary Table S4. MS2E- and the Abbott assay showed no cross-reactivity and gave consistent total 25-OH-D results regardless of increasing 3-epi-25-OH-D_3_ concentrations with mean % biases of 10.6 and 5.0 respectively. The MS methods which do not separate 3-epi-25-OH-D_3_ (MS1 & MS2), and both the Beckman and Roche assays all showed significant cross-reactivity, with increasing degrees of interference correlating with increasing 3-epi-25-OH-D_3_ concentrations. The Siemens assay showed a consistent positive mean bias of ∼14.3% throughout 11 of the 12 samples, but no significant cross-reactivity with 3-epi-25-OH-D_3_.

## 4 Discussion

With vitamin D deficiency being one of the most prominent health problems in Korea [20], accurate 25-OH-D measurements are important to ensure appropriate diagnosis and therapeutic response monitoring. In this multicenter study, we investigated the analytical interference due to endogenous interferents and cross-reactivity with 3-epi-25-OH-D_3_, of common 25-OH-D tests routinely used in Korean laboratories. Two different MS methods and 4 commercial immunoassays for measuring total 25-OH-D were compared, and after proving analytical equivalence (Fig. 1) and recovery (Fig. 2) between MS methods, the mean values of the MS methods were used as reference values to compare analytical interference of the 4 immunoassays. To evaluate interference due to endogenous substances, residual icteric, lipemic, hemolyzed, high RF samples, or samples from myeloma patients or patients undergoing hemodialysis, were used to compare results between the evaluated assays (Fig. 3 and Table 2).

In the present study, consistently significant (>5% in all samples) interference was observed in hemolytic samples (Roche assay), icteric samples (Beckman and Siemens assays) and lipemic samples (all 4 immunoassays) which are common sources of analytical interference in immunoassays [21,22]. In hemolytic samples, the Roche assay consistently gave negative results deviating >5% to MS results, with 3 of 5 samples giving biases of >10%, while other immunoassays also showed several biases >10%. According to manufacturers’ data (Supplementary Table S2), the Roche assay has the highest resistance to hemolysis, which is contrary to our results, although regression data could not prove a relationship with increasing H-index and degree of interference. Due to unavailability of complete blood counts and plasma hemoglobin results, selection of hemolyzed samples involved a visual inspection step with subsequent confirmation via a H-index measured on a Beckman Coulter AU5800 series analyzer. The H-index consists of 6 levels divided according to hemoglobin levels [23], and the absence of samples with a maximum H-index (corresponding to Hb > 500 mg/dL), as well as the small number of evaluated samples, could explain the lack of a significant regression relationship.

Positive biases of 116.2–591.5% and 34.4–497.4% were observed from 25-OH-D measurements of icteric samples of the Beckman and Siemens assay, respectively. To our knowledge, bilirubin interference associated with 25-OH-D measurements has not been reported, although in general, high bilirubin is known to cause spectral interference near absorbance peaks of 456 nm or chemical interference with peroxidase-catalysis [24]. Interestingly, the range (22.5–33.4 mg/dL) of total bilirubin in the present study were all within the 10% bias limit of 40 mg/dL supplied by both Beckman and Siemens.

The negative bias (approximately 20–30%) in 25-OH-D measurements due to lipemia is consistent with previous reports [9,10], whereby lipids interfere with either the light scatter in photometric methods or via volume displacement due to a decreased aqueous phase [24]. Coincidentally, the triglyceride (TG) range (550–1510 mg/dL) used in the present study also did not exceed the 10% bias limit (TG > 3280 mg/dL) provided for the Beckman immunoassay, but a similar negative bias to other immunoassays was observed, nonetheless. The interference due to endogenous substances even at levels within the provided 10% bias limits may warrant re-examination of the stated bias limits.

Large positive biases in 25-OH-D measurements due to paraprotein interference have been reported in previous studies [25,26], due to direct binding to coated microparticles or formation of macrocomplexes. In the present study, the Abbott assay showed the most positive bias (8/10 samples) among the evaluated immunoassays. However multiple regression could not find a significant relationship between both increasing absolute paraprotein levels or relative paraprotein/total protein levels. This may be due to a lack of evaluated samples (only 1 of 10) with total protein levels >12 g/dL, which is the 10% bias limit provided for the Abbott and Siemens assays.

In high RF and hemodialysis samples, the Roche assay, for which a 10% bias limit for RF is not provided, showed a consistent negative bias in 10/10 and 9/10 samples, respectively, although regression could not prove a significant relationship between an increase in RF and the degree of interference. RF can act as a heterophilic antibody [24], and interferences due to RF have been observed in other chemistry immunoassays [27]. Also, in hemodialysis samples, the Siemens assay showed positive biases in all but one sample, and the Beckman and Siemens assays intermittently gave results with biases exceeding >100%. Low 25-OH-D levels are often observed in rheumatoid arthritis [28,29] and chronic kidney disease patients undergoing hemodialysis [30,31], but a reverse association or mechanism with 25-OH-D assays specifically has not yet been reported in literature.

Most of the large % biases (e.g., >100%) observed were from low (<10 ng/mL) 25-OH-D samples, and can be attributed to the fact these concentrations are either near or below the lower limits of quantification of the immunoassays (Table 1). Also, assay imprecision at such low levels would also contribute to the large % biases. Even after consideration of such sensitivity issues, most immunoassays gave results with % biases deviating >10% from MS results. These findings imply a possible underestimation of the vulnerability of immunoassays to endogenous interferents and highlight the lack of, and hereby overall importance of, standardization of 25-OH-D assays to improve overall accuracy.

Interference due to cross-reactivity with 3-epi-25-OH-D_3_, a common 25-OH-D epimer especially in newborns, infants, and pregnant women [[32], [33], [34]], was evaluated via utilizing level 4 NIST SRM 972a standard material (Fig. 4) and serially spiked samples (Fig. 5). Both MS methods (MS1 & MS2) were non-3-epi-25-OH-D_3-_separating methods, thus showing cross-reactivity with 3-epi-25-OH-D_3,_ and gave results within 5% bias limits of the expected value (55.94 ng/mL) of the sum of 25-OH-D_2_, 25-OH-D_3_, and 3-epi-25-OH-D_3_. The Roche assay also showed cross-reactivity, as expected from manufacturers’ data, but the Beckman assay showed less than expected cross-reactivity, giving results slightly above the expected value (29.95 ng/mL) for the sum of 25-OH-D_2_ and 25-OH-D_3_ only. The epimer-separating MS method (MS2E-) was introduced in measurements of serial 3-epi-25-OH-D_3_ spiked samples, and showed no cross-reactivity, contrary to MS1 and MS2 (Fig. 5). Among the immunoassays, the Abbott assay showed the most stable performance with a mean bias of 5.0% over all concentrations, followed by the Siemens assay, which despite a consistent positive bias of ∼14.3% throughout 11 of 12 samples, showed no significant cross-reactivity with increasing 3-epi-25-OH-D_3_ concentrations. In general, cross-reactivity was observed as in accordance to manufacturers’ claims, with the Abbott and Siemens assay showing negligible cross-reactivity, and the Roche and Beckman assay showing significant cross-reactivity. In contrast to 3-epi-25-OH-D_3_, the cross-reactivity with 24,25-(OH)_2_-D_3_ was not evaluated in the present study. 24,25-(OH)_2_-D_3_, which can range from 2 to 20% of total 25-OH-D [35], has been reported to cross-react with ligand-binding assays, particularly in high 25-OH-D samples [11]. In most of our study samples, 24,25-(OH)_2_-D_3_ levels were very low, which can be partially attributed to the rarity of high 25-OH-D level samples, again reflecting the high prevalence of vitamin D deficiency in the Korean population. The 4 levels of NIST SRM 972a also did not contain high levels of 24,25-(OH)_2_-D_3_, and various practical constraints of each participating institute restricted a spiking experiment akin to the present 3-epi-25-OH-D_3_ study. According to manufacturer-provided data (Supplementary Table S3), if 24,25-(OH)_2_-D_3_ levels are sufficiently high, significant cross-reactivity with the Abbott assay, and to a lesser degree, the Roche assay could be expected. A future study with more samples with high 25-OH-D (and consequently 24,25-(OH)_2_-D_3_) could show cross-reactivity with commercial immunoassays.

The clinical implications of 25-OH-D epimers and the associated metabolism of the hydroxyl group are a relatively novel concept [1]. Although all vitamin D metabolites can be epimerized at the C3 position via the 3-epimerase enzyme, the encoding gene has yet to be identified [36]. 3-epi-1,25(OH)_2_-D_3_, the epimer of the biologically active 1,25-(OH)_2_-D metabolite, has fewer calcemic effects than its non-epimer counterpart [37] and both 3-epi-25-OH-D_3_ and 3-epi-1,25(OH)_2_-D_3_ have less affinity with vitamin D binding protein (VDBP) and vitamin D receptor (VDR) than their non-epimer forms, leading to a reduced ability to induce calcium transport [38]. Furthermore, it has been reported that up to 38% of pregnant women and 80% of newborns are classified as vitamin D deficient if 3-epi-25-OH-D_3_ is not included in total 25-OH-D [34]. Due to uncertainty regarding the biological activity of 3-epi-25-OH-D_3_, it is recommended to not include 3-epi-25-OH-D_3_ in total 25-OH-D measurements [11,39], especially in institutes with high numbers of pediatric or obstetric patients [40]. Despite these recommendations, few labs account for 3-epi-25-OH-D_3_ cross-reactivity [1,11], and a recent intercomparison study from the VDSP found that several LC-MS/MS assays from participating laboratories did not separate 3-epi-25-OH-D_3_, leading to significant mean % biases [13]. Likewise, the 2 MS methods in our study (MS1 & MS2) also do not separate 3-epi-25-OH-D_3_ from 25-OH-D_3_. Due to various practical issues, both MS-using institutes use non-epimer-separating MS methods for routine measurements, but both agree that the study results warrant consideration for introduction of an epimer-separating method akin to MS2E-, or a change in LC column(s) and/or protocol, especially when analyzing pediatric or obstetric samples.

There are certain limitations to this study. First of all, the epimer-separating MS2E-method was unavailable to be used as reference values when evaluating endogenous interferents. Due to various issues including maintenance difficulties and lower throughput, the epimer-separating MS2E-method is not routinely used. Furthermore, the sharing of study samples between multiple institutes meant that the available margins of sample volumes were very tight, and eventually there were not enough samples with sufficient volumes to run additional testing with the MS2E-. Secondly, although a multicenter study, the results used for analysis were from single measurements from each institute, each with different methods, thus rendering the results vulnerable to random error. Ideally, duplicate measurements or more, and the same assay tested at multiple institutes, would give more informative results, but difficulties of obtaining samples with sufficient volumes to meet these ideal conditions led to a compromise. Also, the sample number for evaluating endogenous interferents was very small (5 or 10 samples per interferent), which may be the reason for the lack of significant relationships shown between increasing interferent levels and the degree of interference. This limitation was due to constraints with obtaining samples with interferent levels sufficiently high enough to test the 10% bias limits of the commercial immunoassays. Conversely, the fact that these high endogenous interferent conditions are clinically rare could indicate that the current 10% bias limits are adequate, except for bilirubin and lipids, which showed significant interference in 2/4, and all 4 immunoassays, respectively. Furthermore, again due to insufficient sample volumes, plasma hemoglobin values of the hemolytic samples were unavailable, meaning only visual inspection of hemolysis and H-indexes were available for confirmation. Finally, true values for reference samples were only available for the 4 levels of NIST SRM 972 standard material, due to lack of accessibility to a vitamin D RMP. Were true values available for all samples and collected in a prospective manner, a more comprehensive study could be conducted, especially if values of metabolites including 1,25-(OH)_2_-D_3_, free 25-OH-D, bioavailable 25-OH-D (calculable with VDBP), and vitamin D metabolite ratio (calculable with 25-OH-D_3_ and 24,25-(OH)_2_D_3_) are also available [[41], [42], [43], [44]].

In summary, this is the first multicenter study to evaluate the analytical interference due to endogenous interferents and cross-reactivity with 3-epi-25-OH-D_3_ in 25-OH-D assays commonly used in Korean laboratories. Most observed interferences were consistent with bias and cross-reactivity limits supplied by manufacturers. Nevertheless, clinical vigilance is required to detect potential interference, which will ultimately lead to improved clinical outcomes. Moreover, due to the high cross-reactivity of 3-epi-25(OH)D_3_, laboratories should look to change protocols or implement methods that can separate 3-epi-25(OH)D_3_, especially when interpreting vitamin D results of newborns, infants, and pregnant women.

## Funding and acknowledgments

This study was supported and funded by the Korean Society of Clinical Chemistry (KSCC) in 2021.

## CRediT authorship contribution statement

**Joon Hee Lee:** Writing – review & editing, Writing – original draft, Validation, Software, Methodology, Investigation, Formal analysis, Data curation. **Jong Do Seo:** Writing – review & editing, Writing – original draft, Visualization, Methodology, Investigation, Formal analysis, Data curation. **Kyunghoon Lee:** Supervision, Resources, Methodology, Investigation. **Eun Youn Roh:** Supervision, Resources, Investigation. **Yeo-Min Yun:** Supervision, Resources, Project administration, Investigation, Funding acquisition, Conceptualization. **Yong-Wha Lee:** Supervision, Resources, Project administration, Investigation, Conceptualization. **Sung-Eun Cho:** Writing – review & editing, Supervision, Resources, Project administration, Methodology, Investigation, Conceptualization. **Junghan Song:** Writing – review & editing, Validation, Supervision, Resources, Methodology, Investigation, Formal analysis, Data curation, Conceptualization.

## Declaration of competing interest

The authors declare that they have no known competing financial interests or personal relationships that could have appeared to influence the work reported in this paper

## Data Availability

Data will be made available on request.
