# Supplementary material for: Multicenter comparison of analytical interferences of 25-OH vitamin D immunoassay and mass spectrometry methods by endogenous interferents and cross-reactivity with 3-epi-25-OH-vitamin D3
Source: Pract Lab Med. 2023 Dec 12;38:e00347. doi: 10.1016/j.plabm.2023.e00347 (PMC10770599; doi:10.1016/j.plabm.2023.e00347)
Supplement: Multimedia component 1 [file mmc1.docx]

**S1 Table**. Certified Values for NIST SRM 972a

| NIST SRM 972a | ng/mL |
| --- | --- |
| Level 1 |  |
| 25-hydroxyvitamin D2 | 0.54 ± 0.06 |
| 25-hydroxyvitamin D3 | 28.8 ± 1.1 |
| 3-epi-25-hydroxyvitamin D3 | 1.81 ± 0.1 |
| 24R,25-dihydroxyvitamin D3 | 2.66 ± 0.1 |
|  |  |
| Level 2 |  |
| 25-hydroxyvitamin D2 | 0.81 ± 0.06 |
| 25-hydroxyvitamin D3 | 18.1 ± 0.4 |
| 3-epi-25-hydroxyvitamin D3 | 1.28 ± 0.09 |
| 24R,25-dihydroxyvitamin D3 | 1.41 ± 0.05 |
|  |  |
| Level 3 |  |
| 25-hydroxyvitamin D2 | 13.2 ± 0.3 |
| 25-hydroxyvitamin D3 | 19.8 ± 0.4 |
| 3-epi-25-hydroxyvitamin D3 | 1.17 ± 0.14 |
| 24R,25-dihydroxyvitamin D3 | 1.62 ± 0.06 |
|  |  |
| Level 4 |  |
| 25-hydroxyvitamin D2 | 0.54 ± 0.06 |
| 25-hydroxyvitamin D3 | 29.4 ± 0.9 |
| 3-epi-25-hydroxyvitamin D3 | 26.0 ± 2.2 |
| 24R,25-dihydroxyvitamin D3 | 2.64 ± 0.09 |
