# Supplementary material for: Multicenter comparison of analytical interferences of 25-OH vitamin D immunoassay and mass spectrometry methods by endogenous interferents and cross-reactivity with 3-epi-25-OH-vitamin D3
Source: Pract Lab Med. 2023 Dec 12;38:e00347. doi: 10.1016/j.plabm.2023.e00347 (PMC10770599; doi:10.1016/j.plabm.2023.e00347)
Supplement: Multimedia component 2 [file mmc2.docx]

**S2 Table.** 10% bias limits provided by immunoassay manufacturers

|  | Abbott | Beckman | Roche | Siemens |
| --- | --- | --- | --- | --- |
| Bilirubin (mg/dL) | 30 | 40 | 66 | 40 |
| Hemoglobin (mg/dL) | 500 | 50 | 600 | 155 |
| Lipid (mg/dL) | TG, 500 | TG, 3280 | Intralipid, 300 | TG, 540 |
| Biotin (ng/mL) | 30 |  | 30 |  |
| Cholesterol (mg/dL) | 500 | 500 |  | 350 |
| Uric acid (mg/dL) |  |  |  | 20 |
| Total protein (g/dL) | 12 | 6 |  | Ig, 12 |
| Fluorescein (ug/mL) |  |  |  | 0.1 |
| Rheumatoid factor (IU/mL) | 800 | 200 |  |  |
| Goat anti-rabbit ab (μg/mL) | 1 |  |  |  |
| *Abbreviations: TG, triglycerides; Ig, human immunoglobulin | | | | |
