# Supplementary material for: Multicenter comparison of analytical interferences of 25-OH vitamin D immunoassay and mass spectrometry methods by endogenous interferents and cross-reactivity with 3-epi-25-OH-vitamin D3
Source: Pract Lab Med. 2023 Dec 12;38:e00347. doi: 10.1016/j.plabm.2023.e00347 (PMC10770599; doi:10.1016/j.plabm.2023.e00347)
Supplement: Multimedia component 3 [file mmc3.docx]

**S3 Table.** Cross-reactivity (%) data provided by immunoassay manufacturers

|  | Abbott | Beckman | Roche | Siemens |
| --- | --- | --- | --- | --- |
| 25-OH Vit D_3_ | 98.6-101.1 | 100 | 100 | 104.5 |
| 25-OH Vit D_2_ | 80.5-82.4 |  | 93.8 | 100.7 |
| 1,25-(OH)2 Vit D_3_ | 0.1 | 337 |  | 4.0 |
| 1,25-(OH)2 Vit D_2_ | 0 | 1312 |  | 1.0 |
| 24,25-(OH)2 Vit D_3_ | 101.9-189.2 | ~5 | 13.7 |  |
| 24,25-(OH)2 Vit D_2_ | 71.4-114.2 |  |  |  |
| 3-epi-25-OH Vit D_3_ | 1.3 | 65 | 112.9 | 1.1 |
| 3-epi-25-OH Vit D_2_ | 0.8 |  | 91.5 |  |
| Vit D_3_ | 0.8 | 0 | 0.7 | 0.3 |
| Vit D_2_ | 0.4 | 0 | 0.3 | 0.5 |
