# Supplementary material for: Multicenter comparison of analytical interferences of 25-OH vitamin D immunoassay and mass spectrometry methods by endogenous interferents and cross-reactivity with 3-epi-25-OH-vitamin D3
Source: Pract Lab Med. 2023 Dec 12;38:e00347. doi: 10.1016/j.plabm.2023.e00347 (PMC10770599; doi:10.1016/j.plabm.2023.e00347)
Supplement: Multimedia component 4 [file mmc4.docx]

**S4** **Table.** Evaluation of interference due to 3-epi-25-OH Vit D_3_ spiking compared to epimer separating MS results (absolute and percentage differences of MS1, MS2, and immunoassays compared to MS2E- results)

| Epi Spike % | | MS2E- (ng/mL) | Measured 3-epi-D3 (ng/mL) |  |  | MS1 (ng/mL) | (% diff) |  | MS2 (ng/mL) | (% diff) |  | Abbott (ng/mL) | (% diff) |  | Beckman (ng/mL) | (% diff) |  | Roche (ng/mL) | (% diff) |  | Siemens (ng/mL) | (% diff) |  |
| --- | --- | --- | --- | --- | --- | --- | --- | --- | --- | --- | --- | --- | --- | --- | --- | --- | --- | --- | --- | --- | --- | --- | --- |
| 0.0 | | 22.1 | 0.9 |  |  | 23.0 | (4.1) |  | 23.2 | (5.0) |  | 20.3 | (-8.1) |  | 20.2 | (-8.6) |  | 19.9 | (-10.0) |  | 21.3 | (-3.6) |  |
| 10.0 | | 22.0 | 3.0 |  |  | 24.2 | (10.0) |  | 25.3 | (15.0) |  | 19.8 | (-10.0) |  | 24.2 | (10.0) |  | 20.3 | (-7.7) |  | 23.6 | (7.3) |  |
| 25.0 | | 22.5 | 5.1 |  |  | 28.4 | (26.2) |  | 27.0 | (20.0) |  | 19.9 | (-11.6) |  | 22.6 | (0.4) |  | 21.1 | (-6.2) |  | 24.9 | (10.7) |  |
| 50.0 | | 22.3 | 9.3 |  |  | 30.0 | (34.5) |  | 32.7 | (46.6) |  | 19.9 | (-10.8) |  | 23.5 | (5.4) |  | 25.4 | (13.9) |  | 26.1 | (17.0) |  |
|  | |  |  |  |  |  |  |  |  |  |  |  |  |  |  |  |  |  |  |  |  |  |  |
| 0.0 | | 32.4 | 1.6 |  |  | 32.5 | (0.3) |  | 33.0 | (1.9) |  | 31.7 | (-2.2) |  | 31.0 | (-4.3) |  | 30.7 | (-5.2) |  | 37.7 | (16.4) |  |
| 10.0 | | 33.9 | 4.6 |  |  | 37.4 | (10.3) |  | 38.6 | (13.9) |  | 31.3 | (-7.7) |  | 31.6 | (-6.8) |  | 31.4 | (-7.4) |  | 38.5 | (13.6) |  |
| 25.0 | | 33.8 | 8.0 |  |  | 41.4 | (22.5) |  | 40.5 | (19.8) |  | 31.8 | (-5.9) |  | 36.4 | (7.7) |  | 33.9 | (0.3) |  | 37.9 | (12.1) |  |
| 50.0 | | 34.5 | 14.9 |  |  | 46.0 | (33.3) |  | 45.7 | (32.5) |  | 31.3 | (-9.3) |  | 38.7 | (12.2) |  | 40.2 | (16.5) |  | 37.9 | (9.9) |  |
|  | |  |  |  |  |  |  |  |  |  |  |  |  |  |  |  |  |  |  |  |  |  |  |
| 0.0 | | 55.2 | 3.0 |  |  | 53.6 | (-2.9) |  | 58.5 | (6.0) |  | 54.5 | (-1.3) |  | 57.1 | (3.4) |  | 50.2 | (-9.1) |  | 65.0 | (17.8) |  |
| 10.0 | | 55.1 | 7.5 |  |  | 58.7 | (6.5) |  | 65.2 | (18.3) |  | 54.9 | (-0.4) |  | 60.9 | (10.5) |  | 53.1 | (-3.6) |  | 63.7 | (15.6) |  |
| 25.0 | | 54.3 | 13.3 |  |  | 70.2 | (29.3) |  | 65.9 | (21.4) |  | 53.9 | (-0.7) |  | 62.1 | (14.4) |  | 58.1 | (7.0) |  | 64.1 | (18.0) |  |
| 50.0 | | 52.3 | 26.7 |  |  | 80.7 | (54.3) |  | 80.2 | (53.3) |  | 57.0 | (9.0) |  | 68.7 | (31.4) |  | 69.5 | (32.9) |  | 62.2 | (18.9) |  |
|  | *Abbreviations: 3-epi-D_3_, 3-epi-25-OH vitamin D_3_ | | | | | | | | | | | | | | | | | | | | | | |
